# Fenofibrate treatment during withdrawal reverses symptoms of ethanol-induced depression in male rats

**DOI:** 10.3389/fphar.2025.1626031

**Published:** 2025-08-08

**Authors:** Marcelo León, Camila Vásquez-Ulloa, Lucas Marambio-Ruiz, Diliana Pérez-Reytor, Eduardo Karahanian

**Affiliations:** ^1^ Faculty of Health Sciences, Institute of Biomedical Sciences, Universidad Autónoma de Chile, Santiago, Chile; ^2^ Research Center for the Development of Novel Therapeutic Alternatives for Alcohol Use Disorders, Institute of Biomedical Sciences, Universidad Autónoma de Chile, Santiago, Chile

**Keywords:** alcohol use disorder, depression, fenofibrate, neuroinflammation, BDNF, PPAR-α, dendritic arborization

## Abstract

Alcohol use disorder (AUD) and major depression frequently co-occur, both involving significant neuroinflammatory components. Current treatments are often ineffective in addressing AUD-related depression, highlighting the need for novel therapeutic approaches. Previous studies showed that fenofibrate, a peroxisome proliferator-activated receptor alpha (PPAR-α) agonist, reduces voluntary alcohol intake and attenuates neuroinflammation and oxidative stress in alcohol-preferring rats. This study investigated whether fenofibrate administered during alcohol withdrawal could alleviate ethanol-induced depressive symptoms and neurobiological alterations. Male rats received ethanol (1 g/kg, i. p.) on alternate days for 3 weeks; controls received saline. During a 2-week withdrawal period, half of the ethanol-treated rats received fenofibrate (50 mg/kg/day) for the final 5 days. Behavioral assessments included the open field, tail suspension, and sucrose intake tests. RT-qPCR evaluated proinflammatory cytokine and brain-derived neurotrophic factor (BDNF) expression in the prefrontal cortex (PFC) and hippocampus, while Golgi staining assessed dendritic arborization. Ethanol exposure increased anxiety and immobility in behavioral tests, consistent with depressive-like behaviors, and elevated TNF-α, IL-1β, and IL-6 levels. Fenofibrate reversed these behavioral and molecular effects, normalized PFC BDNF expression, and partially restored dendritic complexity. However, ethanol-induced reductions in sucrose intake after withdrawal—reflecting anhedonia—were not reversed by fenofibrate. These findings suggest that fenofibrate mitigates ethanol-induced depressive-like behaviors and neurobiological dysfunctions through anti-inflammatory and neuroprotective mechanisms. Given its established clinical use and safety profile as an FDA-approved drug, fenofibrate shows promise as a translational therapeutic adjunct for treating depression in individuals with AUD.

## 1 Introduction

Alcohol consumption accounts for 4.7% of annual global deaths and for 5.1% of the worldwide disease burden (World Health Organization, 2024). Major depression (MD) ranks as the second leading cause of years lived with disability, contributing to 8% of this parameter at the global level (Vos et al., 2015). Collectively, alcohol use disorder (AUD) and MD contribute to half of the global disease burden caused by mental and substance use disorders (Whiteford et al., 2013). There is a notably high comorbidity between AUD and MD (Brière et al., 2014). Alcohol-related issues in individuals with MD are linked to a more severe course of depression, relapse, heightened risk of suicide and death, and increased healthcare use (Sullivan et al., 2005). Conversely, depressive symptoms frequently occur in AUD, with over one-third of AUD patients meeting the diagnostic criteria for MD at some point in their drinking history (Schuckit, 2006). Compared to those with only one disorder, individuals with co-occurring AUD and MD face a higher risk of alcohol-relapse and dependence, dropping out of treatment, attempting suicide, and experiencing poorer outcomes with antidepressant treatment (Brière et al., 2014; Hasin et al., 2002; Nunes and Levin, 2004).

Research suggests that both AUD and MD, when considered independently, are associated with various immune alterations. However, there is limited understanding of the role neuroimmune function plays in the onset and progression of comorbid AUD and MD. For instance, binge drinking patterns are particularly linked to depression (Boden and Fergusson, 2011), though the precise neurobiological mechanisms underlying this alcohol-induced depression remain unclear. Alcohol significantly impacts the immune system, altering the expression of inflammatory mediators both in the periphery and the central nervous system (CNS). Heavy alcohol use renders the gut wall “leaky”, allowing microbial products like lipopolysaccharides (LPS) to enter the bloodstream (Bala et al., 2014; Enomoto et al., 2000; Keshavarzian et al., 2009; Szabo, 2015). This leaked LPS exacerbates alcohol-induced liver inflammation and activates immune cells, triggering the release of pro-inflammatory cytokines like interleukins IL-1β, IL-6, and tumor necrosis factor-alpha (TNF-α) (Crews and Vetreno, 2016). Cytokines and chemokines produced in the periphery can relay signals to various brain regions, further activating microglia and astrocytes to produce CNS cytokines. The production of these brain cytokines is regulated by NF-κB pathways (Flores-Bastías and Karahanian, 2018). Notably, immune activation in the brain caused by alcohol persists for several months after withdrawal (Qin et al., 2007; 2008). Increased levels of proinflammatory cytokines are observed in the ventral tegmental area, hippocampus, and amygdala in the brains of people with alcohol use disorders (He and Crews, 2008), all of which are critical areas involved in reward, emotion, and behavior. On the other hand, the initial discovery of immune dysfunction in depressive disorders (Maes et al., 1990) gave rise to extensive research that has confirmed that inflammation plays a role in the development and progression of depression. Numerous studies have consistently shown positive associations between MD and inflammatory markers like C-reactive protein (CRP) and IL-6 (Haapakoski et al., 2015; Stewart et al., 2009), IL-1β (Howren et al., 2009), and TNF-α (Dowlati et al., 2010; Y. Liu et al., 2012). Furthermore, cytokine levels in patients with depression have been found to normalize following treatment (Kim et al., 2007).

Several studies have shown reduced serum levels of brain-derived neurotrophic factor (BDNF) in patients with MD compared to healthy individuals (Brunoni et al., 2008; Molendijk et al., 2014; Sen et al., 2008), and evidence also exists to support renormalization of BDNF levels upon successful anti-depression interventions (Brunoni et al., 2008; 2014). BDNF has also been implicated in alcohol dependence and withdrawal (Davis, 2008), and its levels are altered in AUD individuals (Heberlein et al., 2010). After chronic alcohol consumption, BDNF gene expression decreases in the prefrontal cortex (PFC) (Logrip et al., 2009; Melendez et al., 2012). Neuroinflammation and the overexpression of pro-inflammatory cytokines in the CNS can reduce BDNF levels (Flores-Bastías et al., 2020; Guan and Fang, 2006). Thus, decreased BDNF levels are a common factor between MD and AUD, which could be an important cause of the link between the two conditions.

Since neuroinflammation could play an important role in depression triggered by excessive alcohol consumption, the identification of neuroimmune targets to address this condition is highly desirable. In this regard, in recent studies we have shown that the administration of fenofibrate during the withdrawal stage after chronic alcohol consumption in rats reverses ethanol-induced neuroinflammation and brain oxidative stress, along with an 80% decrease in alcohol consumption at relapse (Ibáñez et al., 2023; Villavicencio-Tejo et al., 2021). Fenofibrate is a synthetic agonist that activates peroxisome proliferator-activated receptor alpha (PPAR-α), which negatively regulates NF-κB activity decreasing neuroinflammation (Collino et al., 2006). Another effect of PPAR-α activation is to increase peroxisomal activity of fatty acid degradation, which is why this drug is approved by the FDA and is being used for several years in the clinic for the treatment of dyslipidemia (Cignarella et al., 2006). PPAR-α agonists have been reported in animal models of depression induced by social isolation (B. Jiang et al., 2017), stress (B. Jiang et al., 2015; Ni et al., 2018; Scheggi et al., 2016), and LPS administration (Yang et al., 2017), however the effect of PPAR-α agonists on alcohol-induced depression had not been studied so far. Therefore, given the impact of neuroinflammation in MD, in this work we hypothesized that fenofibrate administration during the withdrawal stage following chronic alcohol consumption in rats will have a positive effect on ethanol-induced depression-associated symptoms.

## 2 Materials and methods

### 2.1 Animals, ethanol administration and fenofibrate treatment

The experiments were performed in 2-month-old male Sprague Dawley rats. Fifteen animals were housed in a temperature-controlled room on a 12-h light/12-h dark cycle, with food and water provided *ad libitum*. Just before starting alcohol treatment, animals were separated into individual cages and baseline levels of depression-associated behaviors were assessed in all of them in this order: open field, sucrose intake, and tail suspension tests -all described in the next section-. The choice of male animals for this study was based on the work of other authors indicating that females are more susceptible to social stress caused by isolation in individual cages, and males are less sensitive to this type of stressor (Palanza et al., 2001). It was reported that individually housed females explored less the unfamiliar area in an open field test and displayed higher risk assessment, a behavioral profile suggestive of higher level of anxiety compared with group-housed females. Additionally, females showed different exploratory behaviors depending on the estrous phase they were in (Palanza et al., 2001). Using male rats, we minimized behavioral changes associated with isolation, and primarily evaluated behavioral changes associated with ethanol exposure.

Since Sprague Dawley outbred rats do not show homogeneous voluntary alcohol consumption (Flores-Bastías et al., 2019; Moorman and Aston-Jones, 2009), we decided to use i. p. administration of ethanol to ensure the same daily dose in all animals. At the start of alcohol treatment, the rats averaged a weight of 403 g (data not shown). Two groups of five rats each (named group 2 and group 3) were administered ethanol 1 g/kg/day via i. p. (30% ethanol solution in saline) on alternate days for 3 weeks. A group of five control rats were injected only with saline i. p. (named group 1). It was reported that intermittent alcohol injection in rats increases neuroinflammation markers and cell death in the cortex and hippocampus, and to produce short- and long-lasting neurobehavioral impairments (Pascual et al., 2007). At the end of the ethanol treatment period (or saline for controls), the animals had gained weight by an average of 7.2%, showing no differences between the three groups (data not shown). The next day after the end of ethanol treatment (or saline for controls), depression-associated behaviors were assessed again in all the animals in the same order described above. Then, a 2-week withdrawal stage began where the group 3 received micronized fenofibrate 50 mg/kg/day resuspended in water (Fibronil, Royal Pharma, Chile) by gavage during the last 5 days of withdrawal (Ibáñez et al., 2023), while the group 2 was administered just water (the vehicle of fenofibrate) by gavage. The saline injected controls (group 1) were also administered water by gavage. At the end of withdrawal, all animals had gained weight by an average of 6.2%, showing no differences between the three groups (data not shown). After these treatments, depression-associated behaviors were assessed in the same order described above for the last time in all the animals (experimental design in Supplementary Figure S1).

The animal study was reviewed and approved by Scientific Ethics Committee and Animal Bioethics, Universidad Autόnoma de Chile.

### 2.2 Behavioral tests

#### 2.2.1 Open field test

This test was used as an index to measure levels of anxiety, which is closely associated with depression (Gould and Dao, 2009). Each animal was placed in an appropriate box measuring 70 cm wide x 55 cm long x 45 cm high, and both average speed when the animals move, and time spent in the center of the box were recorded by a camera in 6-min sessions for each animal. The data were analyzed using Animaze software.

While this test is commonly performed for 10 min, its applicability to shorter durations, such as five or 6 min, has been supported by several recent studies. Gencturk & Unal, (2024) indicate that behavior in this test can be effectively studied in the first 5 min, validating its effectiveness in short periods. Varela et al. (2025) used a 6-min duration in ACTH-treated rats to evaluate changes, highlighting the test’s sensitivity to this time scale. Likewise, Belovicova et al. (2017) pointed out that it is common to evaluate variables such as locomotion, verticality, and grooming in 5-min periods, which allows for accurate and reliable observation of spontaneous behavior. Kalshetti et al. (2015) also applied the test for 5 min in rats, adequately observing the effects of compounds with antidepressant potential. Together, these references support the use of the open field test with 6-min sessions as a scientifically accepted and methodologically solid practice.

#### 2.2.2 Sucrose intake test

Designed to measure anhedonia (M.-Y. Liu et al., 2018), which is a common condition in MD. To measure the basal levels of sucrose intake prior to alcohol treatment, the animals were given a free choice between two bottles for 3 days, one containing 0.2% w/v sucrose and the other containing water. Consumption was recorded day by day and then averaged to calculate basal sucrose ingestion in each animal. This test was repeated after alcohol treatment, and also after fenofibrate treatment, recording sucrose consumption for only 1 day each.

#### 2.2.3 Tail suspension test

This procedure in rodents is conceptually related to the forced swim test, where immobility is induced simply by suspending the animal by the tail in a specially conditioned device. A rat initially tries to escape from tail suspension by performing vigorous movements and then, after a few minutes, become immobile. A shorter time before becoming immobile is related to depressive-like behavior (de Arruda et al., 2022). Although the Tail Suspension Test (TST) was originally developed for mice, several studies have demonstrated its applicability to rats as well, making it a useful and valid tool for assessing depressive-like behaviors in this animal model. For example, this test was used in rats treated with Aβ1-42, using a 6-min suspension protocol, which allowed for an accurate assessment of immobility time, defined as the absence of movement except for breathing and whiskers (Song et al., 2017). Furthermore, Shinde et al. (2015) also used the TST in rats lasting 5 min, which confirms the adaptability of the protocol for this species. According to these antecedents, the mobility of each animal was registered for 6 min of testing.

### 2.3 RT-qPCR for proinflammatory cytokines and BDNF gene expression

At the end of the behavioral tests, the animals were deeply anesthetized with isoflurane and euthanized by decapitation. Brains were extracted, and hippocampus and PFC tissues were dissected from one cerebral hemisphere and immediately homogenized in RNA-Solv Reagent (Omega Biotek, Inc., Norcross, GA, United States) with a mini Potter–Elvehjem pestle (Sigma-Aldrich, St. Louis, MO, United States). Total RNA was extracted according to the manufacturer’s protocols, and RT-qPCR was performed as we previously described (Ibáñez et al., 2023). The primers’ sequences are:

TNF-α (forward) CAG​CCG​ATT​TGC​CAC​TTC​ATA, TNF-α (reverse) TCC​TTA​GGG​CAA​GGG​CTC​TT, IL1-β (forward) AGG​CTT​CCT​TGT​GCA​AGT​GT, IL1-β (reverse) TGT​CGA​GAT​GCT​GCT​GTG​AG, IL6 (forward) CCC​AAC​TTC​CAA​TGC​TCT​CCT​AAT​G, IL6 (reverse) GCA​CAC​TAG​GTT​TGC​CGA​GTA​GAC​C, BDNF (forward) TGA GCC GAG CTC ATC TTT GC, BDNF (reverse) ATA GCG GGC GTT TCC TGA AG, β-Actin (forward) CTT​GCA​GCT​CCT​CCG​TCG​CC, β-Actin (reverse) CTT​GCT​CTG​GGC​CTC​GTC​GC.

### 2.4 Dendritic arborization

To study dendritic arborization we used the classical Golgi staining technique (Dall’Oglio et al., 2008) with some modifications (Zaqout and Kaindl, 2016). For this purpose, the other brain hemispheres were separated and soaked in 20 mL of impregnation solution. After finishing the complete staining protocol, the brains were cut with a vibratome at a thickness of 200–300 μm, collecting slides corresponding to the dorsal hippocampus and PFC on previously gelatinized slides. After the developing protocol (Zaqout and Kaindl, 2016), the slides were mounted with Entellan medium (Merck-Millipore) and photographed by visible light microscopy. The intersections of the dendritic branches of the pyramidal cells of the hippocampus and PFC were traced and quantified by Sholl analysis with Neurolucida software (MBF Bioscience, Williston, VT).

### 2.5 Statistical analysis

**FIGURE 1:**
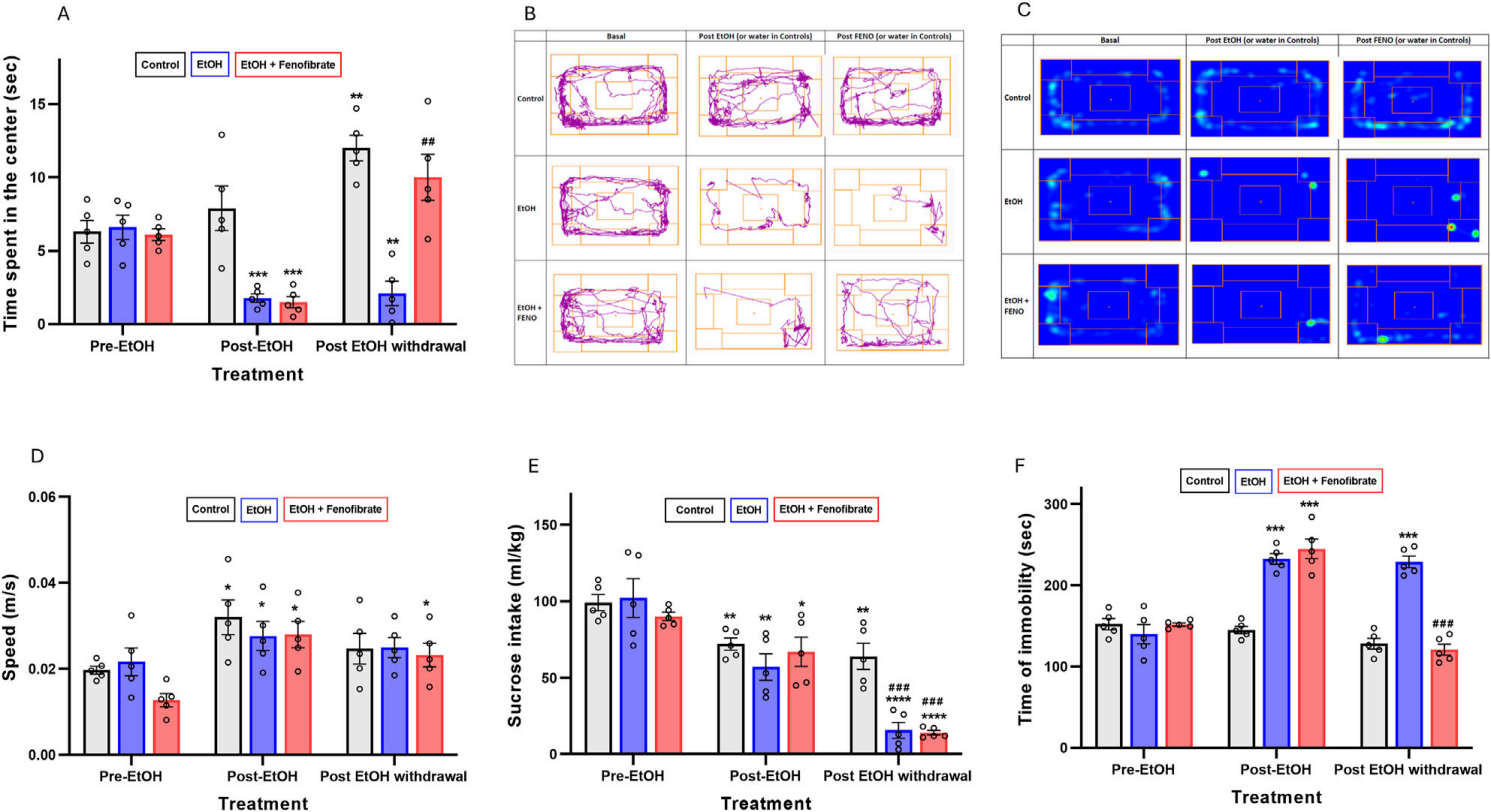
Behavioral tests. **(A)** Open field test. The bars represent the time that the animals spent in the center of the box during the 360 s of the test. **(B)** Open field test. Tracks for a representative animal of each group. **(C)** Open field test. Heat maps for a representative animal of each group. **(D)** Open field test. The bars represent the average speed that the animals reached during their movements. **(E)** Tail suspension test. The bars represent the time that the animals spent motionless during the 360 s of the test. **(F)** Sucrose intake test. The bars represent the 0.2% w/v sucrose intake (mL/kg) in 24 h. All data are presented as mean ± SEM, n = 5 rats per experimental group. Asterisks (*) show statistical significance compared to the pre-EtOH measure of the same group, while hashes (#) show statistical significance comparing the post-EtOH withdrawal and the pre-EtOH measures of the same group. * or #: p < 0.05, ** or ##: p < 0.01, *** or ###: p < 0.001, **** or ####: p < 0.001. One-way multiple measures ANOVA followed by Tukey´s test for *post hoc* analysis.

Statistical analyses were performed using GraphPad Prism 8. Data are expressed as mean ± SEM. For Figure 1, one-way repeated-measures ANOVA applying the Geisser-Greenhouse’s correction (due to low number of subjects), followed by Tukey’s *post hoc* analysis was used. For the rest of the figures, ordinary one-way ANOVA followed by Tukey’s *post hoc* analysis was used. A level of p < 0.05 was considered for statistical significance.

## 3 Results

### 3.1 Behavioral tests

As can be seen in Figure 1, before treatment with ethanol the three groups of animals did not show differences in the time spent in the center (Figures 1A–C) indicating comparable baseline anxiety-like behavior. However, the two groups exposed to ethanol for 3 weeks (post-EtOH) significantly reduced the time spent in the center compared to their respective basal levels (pre-EtOH) (73% and 75% reduction in group 2 and 3, respectively) (one-way repeated measures ANOVA: F_(1.541, 6.163)_ = 60.55 p < 0.01 Geisser-Greenhouse’s epsilon (ε) = 0.77 for group 2; F_(1.001, 4.004)_ = 38.79 ε = 0.50 p < 0.01 for group 3), supporting an anxiogenic-like effect of chronic ethanol exposure. Notably, fenofibrate administration during the withdrawal period restored center time in ethanol-treated animals (group 3) to levels comparable to their own pre-EtOH measures (p = 0.0625) (Figures 1A–C), indicating a potential anxiolytic effect of fenofibrate administered during ethanol withdrawal. On the contrary, in the group treated with alcohol and that did not receive fenofibrate (group 2), the time spent in the center did not change with respect to the measurement in this same group prior to the abstinence stage (post-EtOH) (p = 0.84). As we will discuss below, at the final measurement the control group showed a significant increase in center time relative to their pre-EtOH baseline (ANOVA F_(1.008, 4.031)_ = 49.20 ε = 0.50 p < 0.01). The same trend was also observed in group 3, although the difference was not statistically significant (p = 0.063).

To rule out that the reduced time spent in the center in the ethanol-treated groups was due to musculoskeletal impairments resulting from the alcohol treatment, we quantified the average speed developed by the animals at the moments in which they were actually moving. As observed in Figure 1D, the speed measured on the second occasion (post-EtoH) not only did not decrease but actually increased compared to the baseline levels measured within the same group (pre-EtOH) (one-way repeated measures ANOVA: F_(1.018, 4.072)_ = 13.32 p < 0.05 ε = 0.51 for group 1; F_(1.002, 4.006)_ = 26.23 p < 0.01 ε = 0.50 for group 2; F_(1.016, 4.065)_ = 34.23 ε = 0.51 p < 0.01 for group 3). Since this increase was observed even in the control group without ethanol, we attribute it to a kind of accustoming of the animals to the test box. However, after withdrawal the measured speeds did not change with respect to the post-EtOH measures. These findings would indicate that musculoskeletal impairments did not influence the observed results in the behavioral tests.

Regarding the sucrose intake test (Figure 1E), again the three groups of animals did not show differences prior to ethanol administration. However, the two groups exposed to ethanol (post-EtOH) significantly reduced sucrose intake compared to their respective basal levels (pre-EtOH) (44% and 26% reduction in group 2 and 3, respectively) (one-way repeated measures ANOVA: F_(1.008, 4.033)_ = 131.00 p < 0.001 ε = 0.50 for group 2; F_(1.006, 4.022)_ = 77.90 ε = 0.50 p < 0.001 for group 3), reaffirming the idea that ethanol exposure induces anhedonia. One fact that caught our attention is that the control group not treated with ethanol also lowered its sucrose consumption respect the basal levels (ANOVA: F_(1.108, 4.431)_ = 55.29 p < 0.01 ε = 0.55) -we will comment on this observation in the Discussion section-. After abstinence, the two groups treated with ethanol showed a marked reduction (∼85%) in their sucrose intake compared to their basal levels, regardless of whether they had received fenofibrate or not, which would indicate that abstinence produces a greater increase in anhedonia than exposure to alcohol itself, and that fenofibrate is not able to reverse this effect. In contrast, the 2-week period following saline administration in the controls did not result in a decrease in saccharose consumption in group 1.

As shown in Figure 1F, before treatment with ethanol the animals did not show differences in the immobility time in the tail suspension test. However, after 3 weeks of ethanol administration, immobility time was increased by approximately 60%–65% in groups 2 and 3 (one-way repeated measures ANOVA: F_(1.025, 4.098)_ = 33.72 p < 0.01 ε = 0.51 for group 2; F_(1.087, 4.348)_ = 52.54 ε = 0.54 p < 0.01 for group 3), effect that did not occur in the control group (one-way repeated measures ANOVA: F_(1.040, 4.160)_ = 8.04 p = 0.11 ε = 0.52. However, in the fenofibrate-administered group the immobility time was restored to a value similar to its own baseline before ethanol treatment. In contrast, in the group not treated with fenofibrate (group 2), immobility time remained at a similar value to that prior to withdrawal.

### 3.2 Effect of fenofibrate on ethanol-induced expression of proinflammatory cytokines and BDNF

**FIGURE 2:**
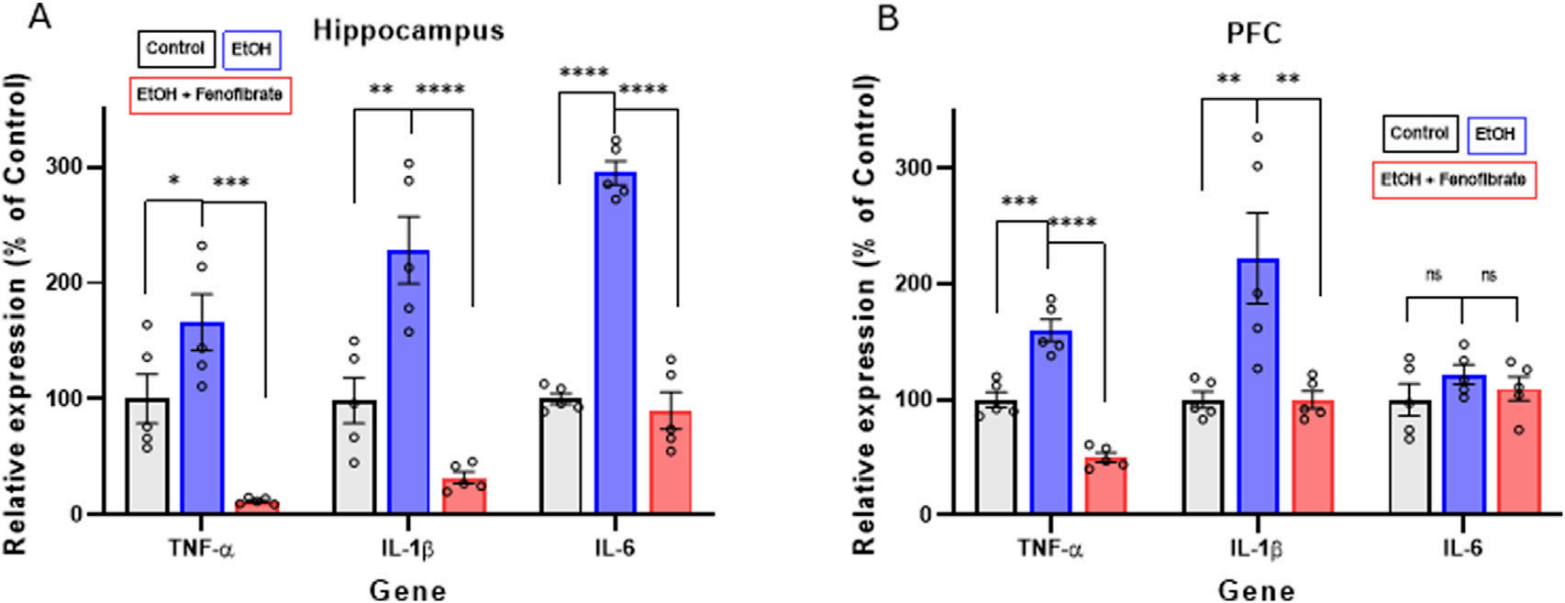
Expression of pro-inflammatory cytokines in hippocampus **(A)** and PFC **(B)**. The graphs show the levels of gene expression as percentages of their control groups, normalized by the levels of expression of β-actin. Data are presented as mean ± SEM, n = 5 rats per experimental group. ** = p < 0.01, *** = p < 0.001, **** = p < 0.0001, ns = non-significant difference between the indicated groups. One-way ANOVA followed by Tukey´s test for *post hoc* analysis.

As we previously reported in other studies (Flores-Bastías et al., 2019; Ibáñez et al., 2023), here alcohol administration also markedly induced the expression of the proinflammatory cytokines TNF-α, IL-1β and IL-6, both in the hippocampus (TNF-α: control 100.0%, EtoH 166.3%, EtOH + Feno 12.1%; ANOVA F_(2, 10)_ = 20.44; p < 0.0001. IL-1β: control 100.0%, EtOH 227.9%, EtOH + Feno 31.9%; ANOVA F_(2, 10)_ = 27.12; p < 0.0001. IL-6: control 100.0%, EtOH 294.6%, EtOH + Feno 90.2% ANOVA F_(2, 10)_ = 135.1; p < 0.0001) and PFC (TNF-α: control 100%, EtoH 159.7%, EtOH + Feno 50.0%; ANOVA F_(2, 10)_ = 73.35; p < 0.0001, IL-1β: control 100.0%, EtOH 222.3%, EtOH + Feno 99.8%; ANOVA F_(2, 10)_ = 10.51; p = 0.0014. IL-6: control 100.0%, EtOH 121.8%, EtOH + Feno 117.6% ANOVA F_(2, 10)_ = 1.689; p = 0.2181) (Figure 2). As we had reported in a recent study (Ibáñez et al., 2023), fenofibrate administration reversed ethanol-induced neuroinflammation, and in some cases cytokine expression was reduced to levels even lower than non-ethanol-treated controls. The only exception to these observations was IL-6 in the PFC, which was neither increased by alcohol treatment nor reduced by fenofibrate treatment.

**FIGURE 3:**
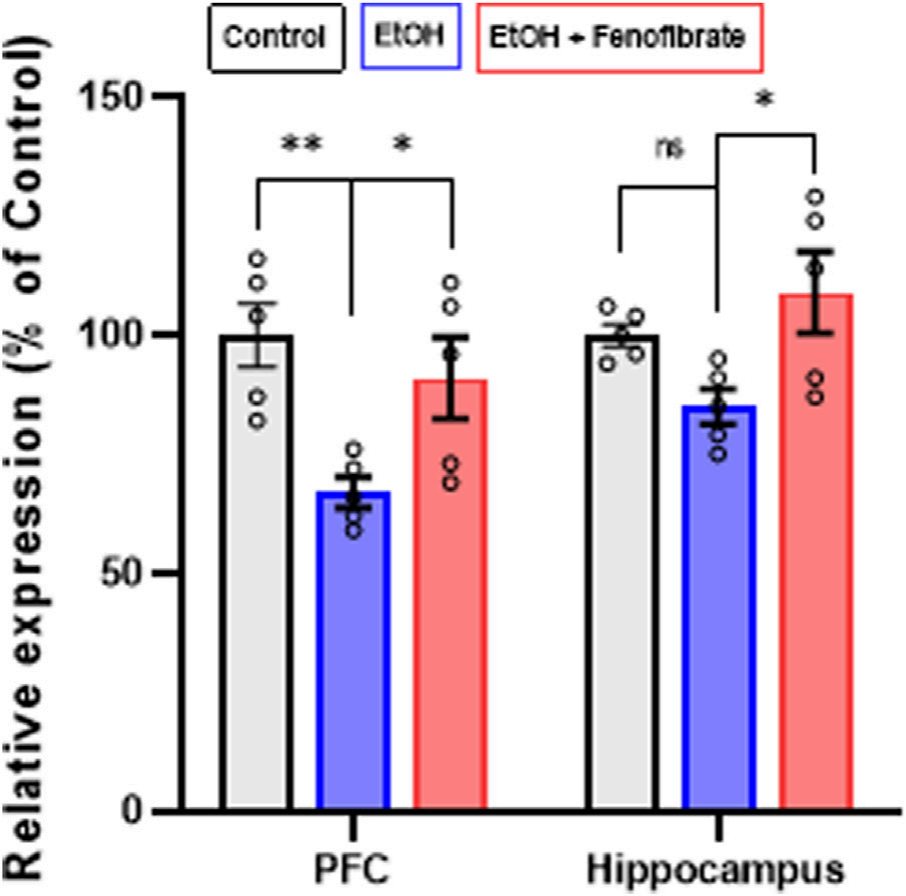
Expression of BDNF in PFC and hippocampus. The graph shows the levels of gene expression as percentages of their control groups, normalized by the levels of expression of β-actin. Data are presented as mean ± SEM, n = 5 rats per experimental group. * = p < 0.05, ns = non-significant difference between the indicated groups. One-way ANOVA followed by Tukey´s test for *post hoc* analysis.

Regarding BDNF expression, Figure 3 shows that ethanol exposure reduced BDNF expression levels in PFC by 33% compared to the non-alcohol treated control group (Control: 100.0%; EtOH: 66.8%; ANOVA F_(2, 10)_ = 6.769; p = 0.0108). A smaller decrease, although not statistically significant, was also seen in the hippocampus of ethanol exposed rats (Control: 100.0%; EtOH: 85.3%; ANOVA F_(2, 10)_ = 4.612; p = 0.1829). Interestingly, fenofibrate treatment increased BDNF expression to values ​​similar to controls in PFC (Control: 100.0%; EtOH + Feno: 91.17%) and hippocampus (Control: 100.0%; EtOH + Feno: 108.7%).

### 3.3 Effect of fenofibrate on dendritic arborization

**FIGURE 4:**
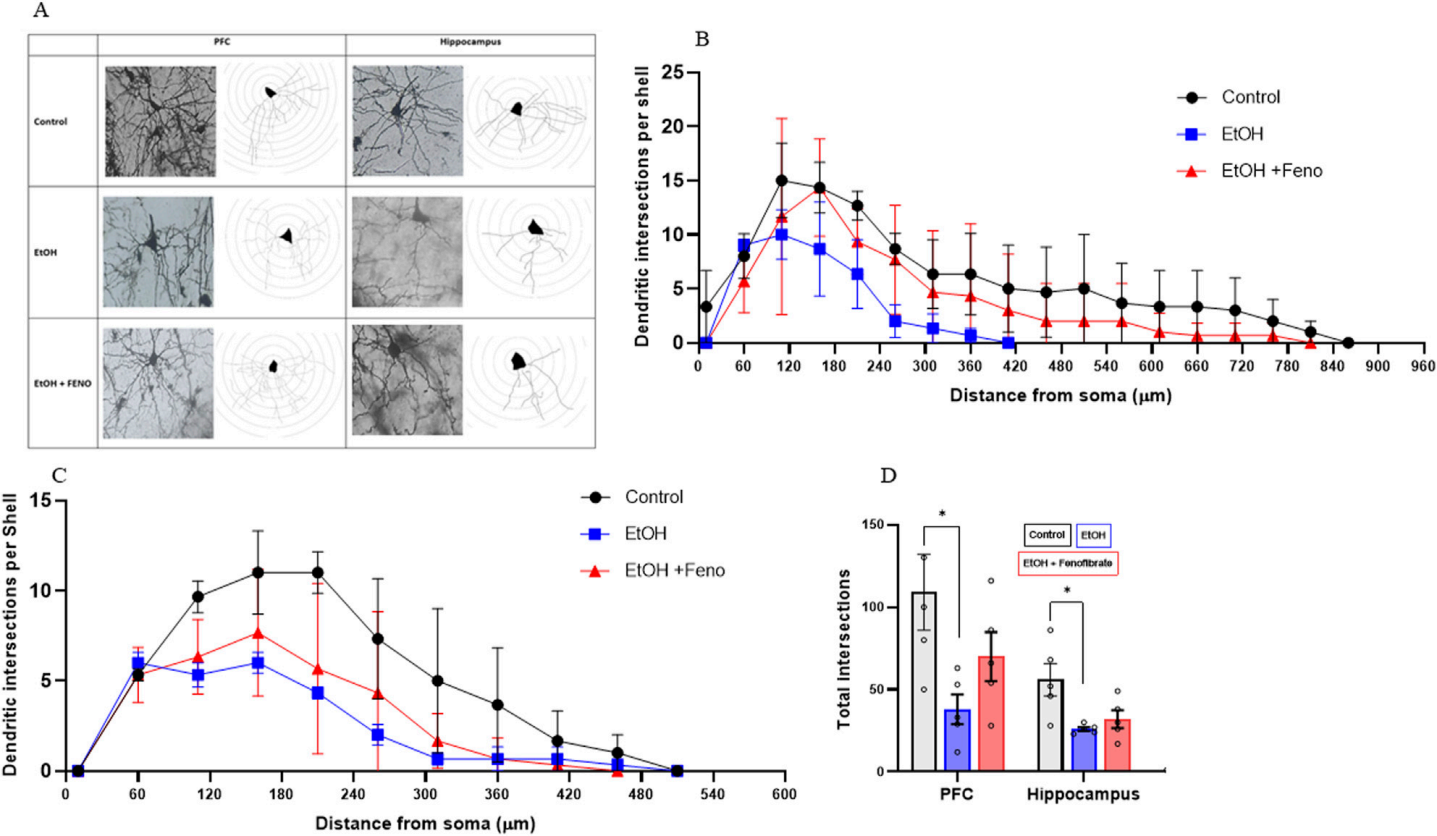
Analysis of dendritic arborization in PFC and hippocampus. **(A)** Micrographs of representative pyramidal neurons of PFC and hippocampus from each group, with their respective Sholl analysis. The number of dendritic intersections is plotted as a function of the distance from the soma in pyramidal neurons of PFC **(B)** and hippocampus **(C)**. **(D)** Total number of intersections determined in the Sholl analysis. Data are presented as mean ± SEM, n = 5 rats per experimental group. * = p < 0.05. One-way ANOVA followed by Tukey´s test for *post hoc* analysis.

As can be seen in Figures 4A–C, the control group showed the highest number of dendritic intersections in pyramidal neurons of the PFC and hippocampus across most distances, peaking around 110–160 µm from the soma. Ethanol group showed a significant reduction in dendritic complexity, with fewer intersections, particularly after 60–110 µm (PFC: ANOVA F_(34, 170)_ = 10.19; p = 0.0035; Hippocampus: ANOVA F_(34, 170)_ = 16.43; p = 0.0003). Fenofibrate treatment appears to partially rescue dendritic complexity compared to ethanol alone, suggesting it has a neuroprotective effect that mitigates some of the ethanol-induced dendritic loss. Additionally, in PFC, a shortening of dendrite length from the soma was observed, to approximately half the length of the control group (Figure 4D) and this shortening was reversed by fenofibrate. Figure 4D shows that, regardless of the distance from the soma, the control group showed the highest number of total intersections in the PFC and hippocampus, and ethanol remarkably reduced dendritic arborization (PFC: control 109; EtOH 38, ANOVA F_(2, 10)_ = 4.267; p = 0.0398. Hippocampus: control 56; EtOH 26, ANOVA F_(2, 10)_ = 5.903; p = 0.0164). Notably, fenofibrate treatment increased the level of dendritic arborization in PFC, but not in the hippocampus (PFC: EtOH + Feno 70; hippocampus EtOH + Feno 32).

## 4 Discussion

Given the high rate of comorbidity between AUD and MD, and that both conditions are themselves strongly related to neuroinflammatory processes, in this study we evaluated the ability of fenofibrate -a drug that we had already demonstrated its anti-inflammatory capacity in the brain of rats treated with ethanol (Ibáñez et al., 2023; Villavicencio-Tejo et al., 2021)- to decrease behavioral symptoms associated with ethanol-induced depression, normalizing BDNF expression and dendritic arborization in the hippocampus and PFC.

In this study, we opted for i. p. administration of alcohol to Sprague-Dawley rats as one of the available methods to ensure that all animals are exposed to the same ethanol dose. In various studies of alcohol-induced depression and anxiety in murine models, other methods have been reported with the same objective: exposure to ethanol vapor (Hartmann et al., 2020; Yao et al., 2021) or exclusive provision of a liquid diet containing alcohol (Huang et al., 2010). In all these studies, the animals developed depressive-like behaviors similar to those reported here, and similar to voluntary consumption studies (see a current review by Mashayekhi-Sardoo et al., 2025).

To assess behavioral symptoms, we first evaluated animals treated with ethanol and fenofibrate using the open field test, designed to measure the levels of anxiety (Gould and Dao, 2009), which is one of the hallmarks of MD. Ethanol treatment for 3 weeks markedly decreased the time that animals spent in the center of the open field box compared to their own basal levels prior to ethanol exposure (Figures 1A–C). We had anticipated this observation, since ethanol treatment and subsequent withdrawal is known to increase the levels of anxiety in both rats and mice (Kliethermes, 2005). It can also be observed in Figure 1A that the control animals gradually increased the time spent in the center on the second and third occasions in which they were subjected to the open field test. This same observation was made (although it does not reach a statistically significant difference) in the fenofibrate-treated group. This could reflect the knowledge and habituation to the test box, which leads to less fear and anxiety in exploring it compared to the first time. Interestingly, fenofibrate administration for the last 5 days of the 2-week withdrawal stage increased time spent in the center to levels that were similar to pre-EtOH measure in the same group. These results would indicate that fenofibrate was able to reverse the anxiety-associated depressive symptoms generated by alcohol administration. Furthermore, the average displacement speeds do not show a reduction due to the ethanol treatment, and even increased post-EtOH, which suggests that treatment with alcohol or fenofibrate does not produce musculoskeletal biases that could account for the observed results (Figure 1D).

In the sucrose intake test, all three groups of rats decreased sucrose consumption in the post-EtOH (or saline for controls) stage compared to their basal sucrose consumption prior to alcohol treatment (Figure 1E). The decrease in the alcohol-treated groups was expected given the known anhedonia generated by alcohol exposure (Boutros et al., 2014; Olney et al., 2018), However, the control group also decreased its sucrose consumption One explanation could be the isolation to which the animals were subjected after the recording of initial basal sucrose consumption; it has been reported that rats raised in individual cages have lower amounts of liking responses to sucrose compared to those raised with environmental enrichment (Wukitsch et al., 2020). Interestingly, both alcohol-treated groups (but not the controls) markedly reduced sucrose consumption after withdrawal compared to the pre-withdrawal stage, regardless of whether they received fenofibrate or not. In this line, there are reports in both humans and animal models that the levels of depression induced by alcohol are even higher during protracted abstinence than immediately after chronic consumption (Oliva et al., 2018; Walker et al., 2010). With these results we can conclude that anhedonia was triggered mainly after withdrawal following alcohol treatment, and that fenofibrate was not able to reverse it in our experimental model. The explanation for why fenofibrate showed no effect in the sucrose intake test is not clear; one possibility is that administration of fenofibrate during only the last 5 days of the 2-week withdrawal phase was insufficient to generate the neurobiological changes associated with sucrose reward (e.g., normalization of the dopaminergic tone in the brain reward circuit). Experiments to assess whether fenofibrate administration normalizes dopamine levels in the nucleus accumbens of rats chronically treated with alcohol are currently underway. These results should be taken into consideration when considering fenofibrate’s translational potential for the treatment of alcohol-induced depression. We believe that longer treatment with fenofibrate during the abstinence period could show better results against ethanol-induced anhedonia.

The third behavioral test corresponded to the tail suspension test, where we obtained results practically identical to those of the open field test (Figure 1F): ethanol treatment increased the time that animals spent immobile hanging by the tail, and fenofibrate reduced immobility time to similar levels as pre-EtOH. This high concordance would somehow validate the results obtained with one or the other test. Our results are consistent with previous reports where ethanol treatment increases immobility time in the tail suspension test in rats and mice, and the administration of anti-inflammatory and antioxidant molecules or proteins reversed this effect of ethanol (Ebokaiwe et al., 2023; X. Jiang et al., 2023).

Regarding proinflammatory cytokine levels, as we had previously reported (Ibáñez et al., 2023), fenofibrate treatment during withdrawal decreased the expression of the proinflammatory cytokines TNF-α and IL-1β in the hippocampus and PFC, while IL-6 decreases only in the hippocampus (Figure 2). The relatively smaller response in the PFC, when compared to the hippocampus may be because the PFC has shown a noticeably lower immunologic reaction to alcohol administration (Villavicencio-Tejo et al., 2021). Similarly, it was reported that ethanol administration does not significantly elevate the expression of pro-inflammatory cytokines such as chemokine C-C motif ligand 2 (CCL2), IL-6, and TNF-α in the mouse cerebral cortex (Kane et al., 2014). Similar to what we reported in a recent study (Ibáñez et al., 2023), for some genes and in certain brain areas, fenofibrate reduced the expression of some proinflammatory cytokines to levels even lower than in controls. We do not have a precise explanation for this consistent observation, but we can attribute it to the potent anti-inflammatory effect of fenofibrate, since it acts by inhibiting the activity of NF-κB (Collino et al., 2006), the master transcription factor for the activation of the innate immune response.

As previously reported in other studies (Logrip et al., 2009; Melendez et al., 2012), in our model ethanol treatment also reduced BDNF expression in PFC (Figure 3). In this work, treatment with fenofibrate during the withdrawal stage fully normalized the expression of this neurotrophin. These results are in line with several studies that reported, in models of depression other than that induced by alcohol, that PPAR-α agonists were able to increase the expression of BDNF (B. Jiang et al., 2015; 2017; Ni et al., 2018; Yang et al., 2017), and that this effect would be directly related to the decrease in neuroinflammation (Yang et al., 2017). Unlike our experimental model, in many of these studies the treatment with the different PPAR-α agonists was performed prior to or during the induction of depressive symptoms so this would limit, in our opinion, the translational options. In contrast, in the studies reported here, fenofibrate treatment was initiated during withdrawal once depressive symptoms had already been induced by alcohol exposure. On the other hand, alcohol treatment slightly decreased BDNF expression levels in the hippocampus, although the difference did not reach statistical significance (Figure 3). Unlike PFC, where decreased BDNF expression by chronic alcohol exposure has been reported (reviewed by (Logrip et al., 2015), the effect on BDNF levels in the hippocampus is still poorly understood. There are even reports indicating that BDNF levels are not reduced by ethanol exposure in this brain area (Darcq et al., 2015; Zhang et al., 2000). However, in the present work fenofibrate treatment still was able to increase BDNF expression in the hippocampus (Figure 3).

The effects of fenofibrate on dendritic arborization (Figure 4) are closely related to its effects in increasing BDNF levels decreased by ethanol exposure (Figure 3). One of the main transcriptional factors involved in dendritic morphology and synaptic plasticity is cAMP response element-binding protein (CREB) (Bibel and Barde, 2000). Since the BDNF signaling pathway culminates in the activation of CREB, this would explain why fenofibrate was able to normalize arborization levels especially in the PFC rather than in the hippocampus. This is especially important, since a strong inverse relationship between severity of depression symptoms and the number of synapses exists (Holmes et al., 2019).

One question that may arise is whether 2 weeks of abstinence could be a sufficiently long period of time to reverse by itself all the effects induced by the previous exposure to ethanol. This did not turn out to be the case, since animals exposed to ethanol and not treated with fenofibrate, after abstinence, maintained the deleterious effects in the behavioral tests, expression of proinflammatory cytokines, and reduction in the expression of BDNF and level of dendritic arborization in PFC. These observations agree with our recent study where 2 weeks of abstinence do not reverse *per se* the severity of relapse, expression of proinflammatory cytokines and levels of oxidative stress in the brain (Ibáñez et al., 2023).

Additionally, we should note that some of the behavioral and molecular changes produced by fenofibrate do not exactly match, as there is a full rescue of the behavioral changes but in some cases the molecular changes are partial or do not occur (e.g., in PFC the normalization of dendritic arborization is not statistically significant, or in hippocampus there are no changes at all (Figure 4). We believe that this may reflect that the rescue of the observed behavioral changes may be the result of a combination of several factors, such as the notable decrease in the expression of proinflammatory cytokines, the normalization of BDNF expression in PFC, etc., so it is feasible that a particular variable analyzed does not show an exact correlation with the observed behavioral variations.

Finally, we acknowledge that we did not include a control group treated only with fenofibrate, which constitutes a limitation of the present study. However, there is evidence that fenofibrate alone does not produce effects on depression-related behaviors; for example, Scheggi et al. (2016) showed that fenofibrate did not modify appetitive motivation for sucrose in rats not exposed to a chronic stress protocol (controls). Fenofibrate is widely used as a lipid-lowering agent in humans, and its adverse effect profile is well documented. Depressive symptoms have not been reported as a frequent or consistent adverse effect in clinical studies or in meta-analyses of fibrate treatments. In databases such as the FDA, EMA, or VigiBase, spontaneous reports of depression as an adverse event associated with fenofibrate use are very rare and anecdotal, and have not been considered statistically significant.

Overall, in this work we provide evidence that would allow glimpse fenofibrate as an adjunct pharmacological therapy for the treatment of depression induced by alcohol abuse. In this sense, fenofibrate has been clinically used worldwide for decades since it was approved in 1994 by FDA for the treatment of dyslipidemia.

## Data Availability

The raw data supporting the conclusions of this article will be made available by the authors, without undue reservation.
