# Supplementary figures and images for: Fenofibrate treatment during withdrawal reverses symptoms of ethanol-induced depression in male rats
Source: Front Pharmacol. 2025 Aug 8;16:1626031. doi: 10.3389/fphar.2025.1626031 (PMC12370632; doi:10.3389/fphar.2025.1626031)

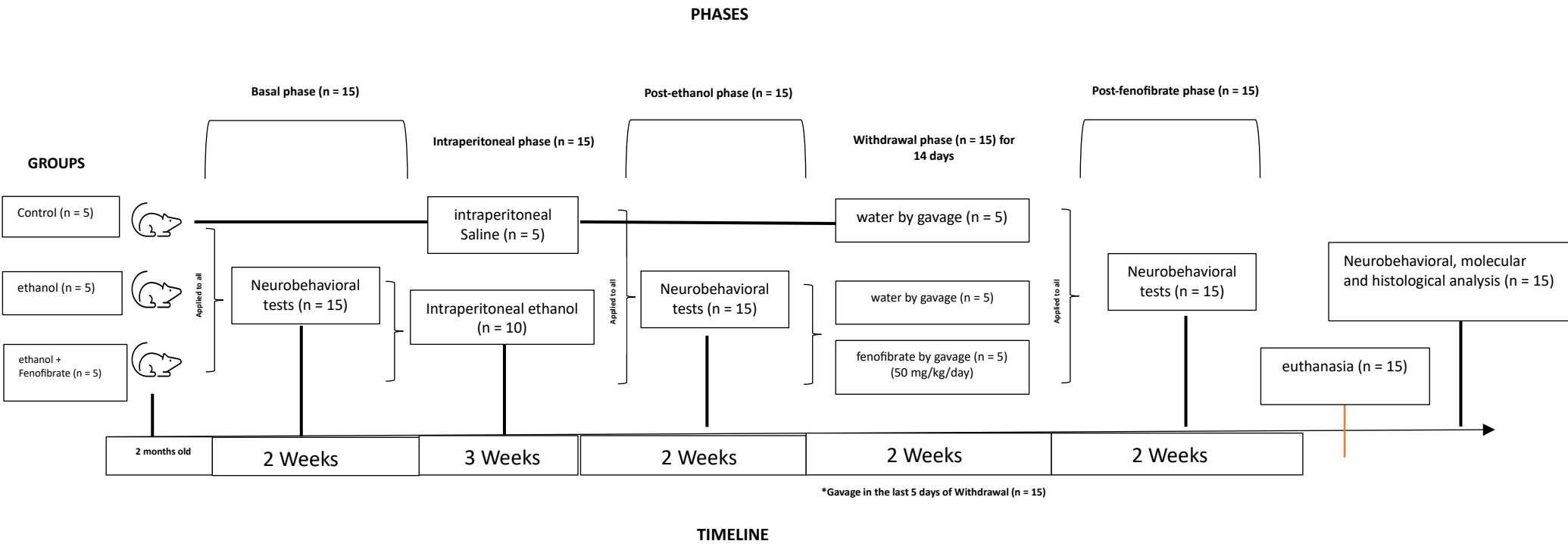

Supplementary Figure 1: Experimental Design

Supplement: Supplementary file 1 [file DataSheet1.pdf]
